# A Review of Animal Models Investigating the Reproductive Effects of Gender-Affirming Hormone Therapy

**DOI:** 10.3390/jcm13041183

**Published:** 2024-02-19

**Authors:** Nicholas S. Raja, Elizabeth S. Rubin, Molly B. Moravek

**Affiliations:** 1 Department of Obstetrics and Gynecology, University of Michigan, Ann Arbor, MI 48109, USA; mpenderg@umich.edu; 2 Department of Obstetrics and Gynecology, Oregon Health & Science University, Portland, OR 97239, USA; rubine@ohsu.edu; 3 Department of Urology, University of Michigan, Ann Arbor, MI 48109, USA

**Keywords:** animal models, gender-affirming hormone therapy, transgender, reproduction, fertility preservation

## Abstract

Gender-affirming hormone therapy (GAHT) is an important component in the process of transitioning for many transgender and gender-diverse (TGD) individuals. Multiple medical organizations recommend fertility preservation counseling prior to initiation of GAHT; however, there remains little high-quality data regarding the impact of GAHT on fertility and reproductive function. A PubMed literature review was performed using Boolean search operators linking keywords or phrases such as “mouse”, “rat”, “primate”, “animal model”, “transgender”, “gender”, “estrogen”, “testosterone”, “fertility”, and “fertility preservation”. Recent research has produced a number of animal models of GAHT that utilize similar hormonal regimens and produce similar phenotypic results to those used and observed in human patients. Specific to testosterone(T)-containing GAHT, animals demonstrate loss of menstrual cyclicity with therapy, resumption of menses on cessation of therapy, suppression of gonadotropin levels, and physical changes such as clitoromegaly. Models mimicking GAHT for transmasculine individuals in the peripubertal period demonstrate that pretreatment with GnRHa therapy does not modify the effects of subsequent T administration, which were similar to those described in adult models. Both models suggest promising potential for future fertility with cessation of T. With estradiol (E)-containing GAHT, animals exhibit decreased size of testicles, epididymis, and seminal vesicles, as well as ongoing production of spermatocytes, and seminiferous tubule vacuolization. Given the ethical challenges of conducting human studies in this area, high-fidelity animal models represent a promising opportunity for investigation and could eventually transform clinical counseling about the necessity of fertility preservation. Future studies should better delineate the interactions (if any exist) between treatment attributes such as dosing and duration with the extent of reversibility of reproductive perturbations. The development of models of peripubertal feminizing GAHT is an additional area for future work.

## 1. Introduction

It is estimated that more than 1.6 million individuals in the United States identify as transgender or gender-diverse (TGD), accounting for approximately 0.5% of the national population [1]. The Human Rights Campaign defines transgender as “an umbrella term for people whose gender identity and/or expression is different from cultural expectations based on the sex they were assigned at birth” [2]. The process of transitioning, or altering one’s gender expression or presentation to align with one’s gender identity, is a deeply personal experience often involving physical or emotional change. Gender-affirming therapies, some of which have the potential to impact future fertility, encompass a wide variety of interventions, including gender-affirming hormone therapy (GAHT), breast or “top” surgery, genital or “bottom” surgery, as well as a range of other urologic, gynecologic, and/or plastic surgeries to alter the face or body [3]. GAHT is pharmacologic hormone blocking or supplementation for the regression or inhibition of current/anticipated undesired secondary sex characteristics and the induction of secondary sex characteristics of a person’s affirmed gender [3]. GAHT generally involves the administration of androgen inhibitors and estradiol (E2) for transfeminine people and testosterone (T) for transmasculine people, though each person’s preferences and goals surrounding transition can be highly individualized [4]. GAHT and, more broadly, alignment of gender presentation with gender identity has been shown to improve quality of life and reduce morbidity for TGD individuals [5,6].

Unfortunately, there remains little high-quality data regarding the impact of GAHT on future reproductive capacity and fertility preservation. This represents a critical gap in our knowledge, as studies indicate that a large proportion of the TGD community is interested in parenthood and/or fertility preservation. Across multiple surveys of transgender adults, 40–67% reported that they desired children in the future, with approximately half of those specifically desiring a genetic connection [7,8,9]. In one study of transgender women, 51% would have considered sperm cryopreservation prior to hormone therapy had it been offered [8]. In a similar study of transgender men, 23% had or would have considered oocyte cryopreservation before transitioning, and 37% would have considered cryopreservation had the technology been available at the time [9]. Consequently, multiple professional organizations, including the American College of Obstetrics and Gynecology, the American Society for Reproductive Medicine, the Endocrine Society, and the World Professional Association for Transgender Health all recommend that transgender individuals receive counseling regarding fertility preservation prior to initiation of hormone therapy [3,5,10]. Unfortunately, gamete cryopreservation can be a physically uncomfortable, time-intensive, and financially taxing process, particularly for oocyte cryopreservation. Moreover, fertility preservation may delay the time to initiating GAHT. As a result, some TGD people begin GAHT without fertility preservation and later express interest in fertility preservation or in utilizing their gametes for family building [7,11].

The paucity of data regarding the impact of GAHT on reproductive function makes it difficult to generate clinical practice guidelines applicable to those currently or recently on hormone therapy. Additionally, available studies have reported conflicting results regarding GAHT-related architectural effects on gonads, impact on ovarian reserve, spermatogenesis, and clinical outcomes related to fertility [11,12,13,14,15,16,17]. Given the ethical challenges of conducting human trials, animal models offer a promising opportunity to better understand the impact of GAHT on reproduction. A PubMed literature review was performed using Boolean search operators linking keywords or phrases such as “mouse”, “rat”, “primate”, “animal model”, “transgender”, “gender”, “estrogen”, “testosterone”, “fertility”, and “fertility preservation”. The intent was to review studies focused specifically on gender care, rather than other conditions such as menopause or polycystic ovary syndrome (PCOS). This work offers a review of existing animal models of GAHT with an emphasis on the reproductive effects of hormone therapy.

## 2. Animal Models of GAHT with Testosterone (T-GAHT) (Table 1)

Most early animal studies investigating the impact of androgen therapy on gonadal architecture and reproductive function were performed in the context of research on polycystic ovarian syndrome (PCOS) [18,19,20]. Several animal models utilizing a range of species, including rodents, sheep, and nonhuman primates, have reported the development of a PCOS-like phenotype with exposure to androgens, including polycystic ovarian morphology with increased antral follicle count, oligo-ovulation, and increased secretion of luteinizing hormone (LH) [18,19]. For a number of reasons, it is difficult to generalize the results of earlier PCOS studies to the GAHT population. In many of these experiments, androgen exposure was begun prior to puberty, significantly earlier than the majority of TGD individuals who begin hormone therapy as adults [3,5,6]. Additionally, the androgen levels, methods, dose, and duration of hormone administration in early animal studies bear little resemblance to current clinical regimens for GAHT.

Two newer models have recently been developed to better mimic modern protocols for hormone administration. Kinnear et al. described a model using adult C57BL/6N female mice injected subcutaneously with T enanthate, an androgen commonly used in human GAHT. Mice were injected at low (0.225 mg), medium (0.45 mg), or high (0.9 mg) doses twice per week for 6 weeks [21]. Apart from one mouse in the low-dose cohort, they found that T-treated mice experienced loss of menstrual cyclicity and persistent diestrus. Serum studies demonstrated persistently elevated total T to physiologic male levels and suppression of LH when compared to controls. Several anatomic changes were also noted in mice receiving T compared to controls, including a significant increase in uterine weight (*p* < 0.05) and enlarged clitoral area (*p* < 0.05). No significant differences in pre-antral follicle count were noted, though T-treated mice showed an increase in atretic late-antral follicles with suppression of corpora lutea (CLs) compared to controls.

A follow-up study by the same group investigated the reversibility of testosterone-induced acyclicity upon cessation of hormone therapy [22]. The authors described a variation on their original model, this time utilizing subcutaneously implanted pellets containing T enanthate, which were subsequently removed after 6 weeks. They reported resumption of normal cyclicity in 100% of mice within 1 week of T cessation. Serum T levels were significantly elevated to physiologic male levels during treatment and fell to comparable levels to controls upon removal. Within 4 cycles of T enanthate implant removal, there were no observable differences between androgen-exposed mice and control mice with regard to serum hormone levels, CL formation, or body morphometrics, with the exception of persistent clitoromegaly in the treatment arm.

The group then conducted another study to better understand the impact of T washout on ovarian dynamics [23]. In this study, C57BL/6N mice were treated with either subcutaneous injections of T enanthate or control sesame oil for 6 weeks. Mice were either sacrificed immediately at 6 weeks (“On T” cohort) or four cycles after the resumption of cyclicity (“Post-T” cohort). Histological analysis revealed multiple changes in the Post-T group. Despite the eventual resumption of cyclicity and return to control serum T levels in all Post-T mice, lower numbers of CLs were observed in this group compared to controls. Follicle distributions were otherwise comparable between groups. Post-T ovaries were also notable for a higher number of large round eosinophilic cells and macrophages. Whole-ovary bulk RNA sequencing revealed significant upregulation of a collection of genes related to immune processes (including regulation of immune response, cytokine production, and regulation of leukocyte activation) in the Post-T group that was not seen in either the On-T or control groups. Based on these results, the authors speculated that T exposure increased the stromal immune response, leading to suppression of CL formation, and that this immune response may be reversible with increased time off T.

Another model was generated by Bartels et al. using adult Hsd:NSA (CF-1) female mice injected subcutaneously with 400 µg T cypionate once weekly for 6 weeks [24]. Similar to Kinnear et al. [21], T-treated mice experienced loss of cyclicity and persistent diestrus that reversed following the cessation of injections and a 6–7 week washout. They likewise reported significantly elevated T levels, falling within the normal range for adult male mice. Anatomically, the authors noted a significant decrease in ovarian weight compared to controls that persisted after washout, though follicle numbers did not differ significantly. They noted similar numbers of antral follicles between T-treated and control mice with almost no atretic follicles in either group; however, there was a significantly higher number of CLs in the control group. Clitoromegaly was also noted in the T-treated group, similar to results by Kinnear et al. [21].; interestingly, however, clitoromegaly was no longer apparent in these mice following a washout period [21].

**Table 1:** Animal models of T-GAHT.

Study	Strain and Age	Hormone Treatment	Exposure and Washout Durations	Control	Experimental Design	Findings
Kinnear et al., 2019 [21] Female mouse	C57BL/6N 8–9 weeks	T enanthate Subcutaneous injection 0.225, 0.45, or 0.90 mg twice weekly	6-week exposure	Sesame oil vehicle injections	Immediate post-treatment assessment of hormone profile, cyclicity, phenotypic changes, ovarian histology.	- Loss of menstrual cyclicity - ↑ T to physiologic male levels - Suppression of LH - ↑ Uterine weight and clitoral area - No differences in pre-antral follicle count - ↑ Atretic late-antral follicles - Suppression of CLs
Kinnear et al., 2021 [22] Female mouse	C57BL/6N 9–10 weeks	T enanthate Subcutaneous pellet implant 10 mg	6-week exposure 4-cycle washout	Sham pellet	Assessment of hormone profile, cyclicity, phenotypic changes, ovarian histology post-treatment and after washout.	- ↑ T to physiologic male levels - Washout: - Resumption of cyclicity within 1 week - Return to control T levels - Within 4 cycles: No differences in hormone levels, CL formation, or non-genital body morphometrics
Bartels et al., 2021 [24] Female mouse	Hsd:NSA (CF-1) 6 weeks	T cypionate Subcutaneous injection 400 ug weekly	6-week exposure 6–7-week washout	Sesame oil injection	Assessment of hormone profile, cyclicity, phenotypic changes, ovarian histology post-treatment and after washout.	- ↑ T to physiologic male levels - Loss of cyclicity - ↓ Ovarian weight - No difference in follicle count Washout: - Resumption of cyclicity - Return to control T levels - Persistent ↓ ovarian weight

T: testosterone, LH: luteinizing hormone, CL: corpus luteum.

## 3. Animal Models of Fertility Preservation Procedures in the Setting of T-GAHT

As very little is known regarding the impact of long-term T therapy on subsequent fertility preservation efforts, transmasculine people have historically been recommended to discontinue T-GAHT for some length of time prior to controlled ovarian hyperstimulation [11,25]. However, there are no current guidelines advising clinicians on how long T-GAHT should be stopped, and, in fact, it remains unknown if cessation is necessary at all [5]. Both of the previously mentioned mouse models have been used to investigate in vitro fertilization (IVF) outcomes in the setting of T therapy.

In the second phase of their study, Bartels et al. established two cohorts of mice, an “active exposure” group of mice sacrificed immediately after the final T injection and a “washout” group sacrificed 6–7 weeks after the final T injection [24]. Both cohorts had their own control groups with identical sacrifice timing but were injected with placebo sesame oil only. Each of these cohorts was then divided into three treatment subgroups: mice stimulated with equine chorionic gonadotropin (eCG) only, mice stimulated with eCG followed by ovulation induction with human chorionic gonadotropin (hCG), and mice that were not stimulated. After treatment, bilateral salpingo-oophorectomy was performed. In the unstimulated and eCG-primed groups, the ovarian cortex was punctured, and oocytes were aspirated and counted before undergoing in vitro maturation (IVM). In the ovulation induction (eCG + hCG) group, cumulus masses were aspirated from the oviducts, mixed with sperm extracted from the epididymides of male mice, and two-cell embryos were counted following incubation. The authors then analyzed oocyte number, meiotic spindle structure following IVM of immature oocytes, and mature oocyte fertilization rates and two-cell embryo formation.

They found that T-treated ovaries responded to treatment, with greater numbers of preovulatory follicles in the stimulated group compared to unstimulated. Analysis of immature oocytes dissected from stimulated and unstimulated mice demonstrated significantly more immature oocytes from ovaries of T-treated active exposure mice compared to controls, though the ovulation induction in “active exposure” and washout groups ovulated similar numbers of oocytes. Additionally, the proportion of immature oocytes with morphologically normal meiotic spindle formation was similar between all groups. Among mature oocytes, rates of fertilization and development of two-cell embryos were comparable between the active-exposure T, washout T, and control groups.

A subsequent study by Schwartz et al. used the aforementioned implant model and similarly divided mice into “current T” and “T washout” groups with matched controls, with 2 weeks of T cessation in the washout cohort [26]. In this study, they also assessed the impact of duration of hormone therapy by dividing the hormone-exposed groups into short- and long-term exposure groups with implants in place for either 6 or 12 weeks, respectively. Each group was then stimulated with intraperitoneal injections of HyperOva (Cosmo Bio Ltd., Tokyo, Japan) and underwent ovulation induction with hCG with subsequent fertilization of retrieved oocytes. Whereas Bartels et al. [24] examined only two-cell embryos, Schwartz et al. [26] randomized fertilized eggs to embryo transfer at the cleavage stage vs. continued culture through blastulation. 

Compared to controls, the authors found that both short- and long-term current T exposure were associated with significantly lower oocyte yield, translating to lower numbers of mature oocytes, cleavage-stage embryos, and blastocysts. However, it is important to note that rates of maturity, fertilization, and blastulation were not different compared to controls. The short-term T exposure group experienced recovery following washout, with no difference in two-cell embryos compared to controls. In contrast, only partial recovery was noted among the long-term T-exposure group following washout. Live birth rates (LBRs) following cleavage-stage embryo transfer were not different between groups regardless of washout, although a trend toward lower LBR was noted in the long-term exposure group (6.7% vs. 22.6%, *p* = 0.0573). Their study was not powered to examine LBR as a primary outcome.

Taken together, these studies suggest that ovaries from T-exposed mice do respond to stimulation and produce normal, fertilizable oocytes and embryos (Table 2). Their data conflict regarding the impact of washout on oocyte retrieval following ovulation, with one study suggesting a benefit to washout and another suggesting no benefit. Furthermore, the study by Schwartz et al. [26] suggests that a longer duration of T-GAHT may inversely correlate with oocyte yield, even after washout. Notably, aneuploidy, a major contributor to IVF inefficiency, has not yet been evaluated in any of these models. Additional investigation is needed to understand the functional and clinical consequences of observed changes in ovarian histology following cessation of T, as well as to better understand the impact of hormone therapy on oocyte retrieval and LBR. 

## 4. Animal Models of GAHT with Estradiol (E-GAHT)

Compared to T-GAHT, animal data regarding the reproductive impact of E-GAHT in transfeminine people are even more sparse (Table 3). Similar to studies on androgen exposure, early animal models examining estrogen therapy in male rats are likely not translatable to the GAHT population—often with early hormone exposure, use of castrated rats, or treatment regimens that bear little resemblance to modern gender-affirming care. GAHT protocols for transfeminine people involve estrogen therapy commonly accompanied by antiandrogens [4,6]. While models have been created to examine the impact of feminizing GAHT on hormone profiles, spermatogenesis, and reproductive tissues, to our knowledge, no animal studies have been performed with the specific intent of assessing functional fertility outcomes after feminizing hormone therapy.

Two recent animal models have been developed utilizing estradiol (E2) monotherapy. Alexander et al. presented a model of male Sprague Dawley rats implanted with subcutaneous capsules containing 17-beta E2 benzoate at low (2.5 mg), medium (5 mg), and high (7.5 mg) doses [27]. They reported physiologic female levels of serum E2 with all three doses following 3 weeks of therapy and found that administration of E2 was associated with a significant reduction in testicular weight vs. controls (*p* < 0.001). Pfau et al. utilized adult male C57BL/6NHsd mice who received subcutaneous implants containing either 1.25 mg, 2.5 mg, or 5 mg of estradiol powder or a control capsule [28]. All implanted capsules were left in place for 6 weeks. They reported suppression of serum T and follicle-stimulating hormone (FSH) levels accompanied by elevation of E2 to the physiologic levels of female mice at all implant doses; however, only the 5 mg group demonstrated suppression of LH. E2 administration also induced various anatomical changes in this model. Similar to Alexander et al. [27], they noted a significant decrease in testicular weight in mice receiving E2. Additionally, all E2-treated mice experienced a decrease in the size of the epididymis and seminal vesicles. Histologically, the moderate- and high-dose E2 treatment groups had significantly higher numbers of vacuolitic Sertoli cells, a marker of germinal cell degeneration or atrophy. No significant morphological changes were noted in Leydig cells in response to E2 treatment. While all groups produced both immature and mature spermatocytes, no spermatocytes in any of the E2-treated groups demonstrated normal motility but rather exhibited “uncoordinated shaking”. While most GAHT regimens for transfeminine people utilize a combination of estrogen therapy and antiandrogens, these studies are helpful in isolating the impact specifically of estrogen administration in this population.

Two groups have investigated the impact of estrogen therapy in combination with antiandrogens on reproductive parameters in rat models. Tassinari et al. utilized Sprague Dawley rats that were administered subcutaneous injections of 17-beta E2 valerate plus cyproterone acetate in one of three doses five times per week for 2 weeks [29]. Their group similarly reported decreased serum T and increased E2 levels in treated animals, with accompanying decreases in testicular and epididymal weight as well a marked decrease in sperm counts at all doses. Unfortunately, toxicological studies suggested that the selected doses of hormones used were too high to implement the model and likely limited the direct translatability of these results to other animal studies or clinical medicine. Another study by Gusmão-Silva et al. treated adult male Wistar rats with a combination of estradiol enanthate and dihydroxyprogesterone acetophenide (E2EN/DHPA) injected every 10 days for 5 months [30]. They demonstrated testosterone/estrogen ratios similar to female rats following treatment but otherwise did not investigate other impacts on reproductive hormones or tissues.

## 5. Animal Models of GAHT in the Peripubertal Population (Table 4)

Puberty is the developmental period of sexual maturation, during which individuals manifest secondary sex characteristics and gain the ability to reproduce. The initiation of puberty is driven by the pulsatile release of gonadotropin-releasing hormone (GnRH) from the hypothalamus, which stimulates the release of gonadotropins from the pituitary gland and the eventual production of sex hormones by the gonads. It is increasingly recognized that an individual’s sense of gender identity begins to solidify at a very young age. A recent study estimates that there are approximately 300,000 TGD youth in the United States [1]. For many TGD adolescents, the pubertal transition can be accompanied by significant gender dysphoria, as endogenous hormone production drives gender-incongruous secondary sexual characteristics. Thus, the current best practice in care for TGD youth often includes suppressing endogenous hormones for the purpose of interrupting, delaying, or completely avoiding incongruous puberty via therapy with GnRH agonists (GnRHa) or “puberty blockers” [3,4,5,6,31]. GnRHa medications act at the level of the hypothalamus, causing a downstream decrease in sex steroid hormone production at the gonads [31,32]. At an age-appropriate time, GAHT with T or E2 can then be slowly started to induce gender-congruent secondary sexual characteristics. As these youth may never complete gonadal maturation, their future reproductive potential is not well understood. GnRH agonists have been used for decades to suppress precocious puberty and have a well-documented safety profile; [32] however, there is almost no animal or human data regarding the long-term reproductive outcomes for individuals with a history of peripubertal GnRHa use followed by initiation of GAHT.

**Table 4:** Animal models of GAHT in the peripubertal population.

Study	Strain and Age	Hormone Suppression and Treatment	Suppression, Treatment, and Washout Durations	Control(s)	Experimental Design	Findings
Dela Cruz et al., 2023 [33] Female mouse	C56BL/6N 26 days	Depot-GnRHa Subcutaneous pellet implant 3.6 mg T enanthate Subcutaneous injection 0.45 mg weekly	Suppression: 21 days Treatment: 6 weeks	Suppression placebo: Sham surgery Treatment placebo: Sesame oil injection	Evaluation of GnRHa + T vs. GnRHa-only vs. T-only Assessment of hormone profile, cyclicity, phenotypic changes, ovarian histology after discontinuation of GnRHa treatment and after completion of T treatment	Suppression: - Persistent diestrus - ↓ FSH/LH - Post-suppression - ↓ Primary follicles - No differences in primordial follicles between groups - GnRHa-only—Resumption of cyclicity - GnRHa + T and T-only - ↑ T to physiologic male levels - ↑ Ovarian weight; ↓ uterine gland weight and preputial gland weight
Dela Cruz et al., 2023 [34] Female mouse	C56BL/6N 26 days	Depot-GnRHa Subcutaneous pellet implant 3.6 mg T enanthate Subcutaneous pellet implant 10 mg	Suppression: 21 days Treatment: 6 weeks Washout: 2 weeks	Suppression placebo: Sham surgery Treatment placebo: Sham pellet	COH + hCG ovulation induction. IVF and culture to blastocyst stage or transfer at cleavage stage. Comparison of outcomes for GnRHa + T vs. GnRHa-only vs. T-only	COH + IVF after suppression: - GnRHa-only similar to negative controls COH + IVF after T treatment: - ↓ Oocyte yield and blastocyst number - GnRHa + T outcomes were similar to T-only COH + IVF after washout: - Recovered oocyte and blastocyst yield
Godiwala et al., 2023 [35] Female mouse	Hsd:NSA (CF-1) 3 weeks	Depot-LA Intraperitoneal 100 μg every 4 weeks Testosterone cypionate 200 ug or 400 ug weekly	Suppression: 12 weeks Treatment: 8 weeks 4-week overlap	Suppression placebo: PBS injection Treatment placebo: Sesame oil injection Stimulation placebo: Unstimulated	eCG COH + IVM or eCG COH + hCG ovulation induction. IVF and culture to 2-cell stage. Comparison groups of control-only, T-only, or LA-only.	- ↓ Ovarian weight in LA-only and LA + T groups - IVM: oocytes from unstimulated LA + T had lower normal meiotic spindle rates compared to unstimulated T-only and stimulated LA + T and T-only. - eCG/hCG: LA-only and T-only ovulated, only 86% of LA + T - No significant differences in oocyte yield, live birth, offspring sex, or offspring fertility between all groups

GnRHa: gonadotropin-releasing hormone agonist, FSH: follicle-stimulating hormone, LH: luteinizing hormone, IVF: in vitro fertilization, COH: controlled ovarian hyperstimulation, eCG: equine chorionic gonadotropin, hCG: human chorionic gonadotropin, PBS: phosphate buffered saline, IVM: in vitro maturation.

Dela Cruz et al. recently developed a model using peripubertal 26-day-old C56BL/6N female mice implanted with a depot-GnRHa pellet (3.6 mg) for 21 days, followed by weekly T enanthate injections (0.45 mg) for 6 weeks [33]. They evaluated GnRHa + T versus GnRHa-only, T-only, and controls using sham surgeries and vehicle injections as control therapies. The authors found that GnRHa-exposed animals remained in a state of persistent diestrus and acyclicity, with reduced gonadotropin levels compared to controls (FSH *p* = 0.0013 and LH *p* = 0.0002) and a lack of CL formation at the end of the 21-day GnRHa administration period. Following cessation of GnRHa treatment, the GnRHa-only control cohort quickly regained cyclicity. In contrast, the GnRHa-T group remained acyclic and in dietrus throughout the duration of the study. Serum T levels were significantly higher in T-treated mice compared to controls and similar to those of age-matched male mice. Compared to controls, both GnRHa + T mice and T-only mice demonstrated a significant decrease in ovarian weight (*p* < 0.0001 for both groups) and an increase in uterine gland weight (*p* < 0.0001 for both groups) and preputial gland (glands surrounding the clitoris) weight (*p* < 0.0001 for both groups) compared to GnRHa-only and control mice. Interestingly, they also found that the number of primary follicles in the GnRHa + T, GnRH-only, and T-only groups was significantly lower than the control group (*p* < 0.0001, *p* = 0.008, and *p* = 0.0003, respectively), with no differences seen in the number of primordial follicles between groups.

Subsequent work by Dela Cruz et al. utilized a mouse model to investigate the impact of prepubertal GnRHa with T-GAHT on IVF outcomes [34]. Following 21 days of GnRHa or sham treatment, mice were implanted subcutaneously with either T 10 mg or placebo for 6 weeks. After 6 weeks, one cohort of mice immediately underwent superovulation and IVF while a second group had a 2-week washout period after implant removal prior to superovulation and IVF. They then assessed oocyte yield, oocyte maturity rate, fertilization rate, and numbers of two-cell, four- to eight-cell, morula-stage, and blastocyst-stage embryos as well as hatching blastocysts. Consistent with the findings from Schwartz et al. [26], they found decreased oocyte yield and subsequent blastocysts in mice who underwent superovulation at the end of T treatment that recovered after washout. Pretreatment with GnRHa did not appear to modulate the effect of T in IVF outcomes, as mice who received sham followed by T had similar outcomes as those that received GnRHa prior to T. Mice who received GnRHa followed by a placebo implant had similar outcomes to negative controls. Live birth was not assessed.

Expanding upon the Bartels et al. CF-1 mouse model, Godiwala et al. also recently developed a model of puberty suppression followed by stimulation [35]. In this model, 3-week-old female mice were injected intraperitoneally with 100 µg of the GnRHa depot leuprolide acetate (LA) or vehicle (PBS) every 4 weeks for three doses (8 weeks total until 11 weeks). With the third dose of LA, mice received weekly T injections for 4 weeks of 200 µg, followed by 4 weeks of 400 µg, in order to mimic the gradual increase in T dosing typically seen in adolescent gender-affirming care. Compared to the experimental LA-T group, three control mice cohorts were administered vehicle only every 4 weeks for four doses (control), LA only every 4 weeks for four doses (LA only), and control for two doses followed by the low- to high-dose experimental T protocol (T only). Each cohort was then either unstimulated, eCG-stimulated, or eCG-stimulated followed by hCG-induced ovulation. As in the group’s prior study, immature oocytes were collected from unstimulated and eCG-stimulated mouse ovaries for in vitro maturation (IVM), and oocytes were collected from the oviduct in the eCG stimulation with hCG ovulation group. Mature ovulated oocytes underwent IVF with assessment of the rate of fertilization, two-cell embryos, and blastocysts. In three experiments, cleavage-stage embryos were transferred to pseudopregnant CD-1 females to assess live birth and subsequent pup fertility. 

Godiwala et al. [35] also found that the LA- and LA-T-treated mice had lower ovarian weight in their model than control mice, consistent with Dela Cruz et al. [33] Moreover, they noted the ovarian tissue to be subjectively more fragile and prone to deterioration. Additionally, IVM oocytes from unstimulated LA + T-treated mice had significantly lower normal meiotic spindle rates compared to unstimulated T-only and stimulated LA-T and T-only groups. In ovulation IVF studies, all LA-only and LA + T mice ovulated with 10 IU eCG/10 IU hCG dosing, compared to only 12/14 (86%) of mice treated with LA + T. Of note, this dosing was higher than in this group’s prior model; only 62.5% of 9-week LA-treated mice ovulated at the initial 5IU eCG/5 IU hCG in preliminary studies. In contrast to Dela Cruz et al. [34], in ovulation IVF studies, there were no statistically significant differences in the number of oocytes ovulated. Additionally, no differences in live birth, offspring sex, or offspring fertility were noted. 

These studies both suggest that ovaries from puberty-suppressed T-treated mice (GnRHa-T) will respond to gonadotropins though may need high gonadotropin dosing. The oocytes from puberty-suppressed T-treated mice can also lead to normal offspring, even without a washout period. However, data conflict regarding whether continuation of T leads to lower oocyte numbers compared with controls. Moreover, it is not clear that both models completely halted pubertal development and/or ovulation, particularly at lower doses.

## 6. Discussion

Due to the relatively increased logistical and ethical challenges of conducting human studies, high-fidelity animal models that utilize similar hormonal agents and produce similar findings to available human data offer a convenient platform from which to launch subsequent investigations on GAHT. Each model of T-GAHT presented was created with the goal of mimicking human treatments [4,5,6]. In addition, they reproduced several phenotypic findings observed in humans receiving T-GAHT, including the loss of menstrual cyclicity with therapy, resumption of menses on cessation of therapy, suppression of gonadotropin levels, and physical changes such as clitoromegaly. While these findings suggest translatability to human studies, the authors also noted several changes that differ from human data, including increased uterine weight and reversal of clitoromegaly following cessation of T. 

Both models investigating estrogen monotherapy were able to achieve physiologic estrogen levels for female mice. Additionally, their data reproduced multiple findings from human studies, including the decreased size of testicles, epididymis, and seminal vesicles, as well as ongoing production of spermatocytes, and seminiferous tubule vacuolization [36,37,38]. While these findings suggest the translatability of subsequent research to human subjects, most patients undergoing feminizing GAHT receive both estrogens and anti-androgens. Unfortunately, available models of combination therapy are more limited, as one utilized potentially unsafe doses of hormones and the other did not investigate the reproductive impacts of hormone treatment.

To our knowledge, the studies by Dela Cruz et al. [33] and Godiwala et al. [35] are the only two animal models available that simulate GAHT for transmasculine individuals in the peripubertal period or investigate fertility outcomes in this population. Additionally, we could not find any studies mimicking peripubertal GAHT for transfeminine individuals. Animal models focused on the peripubertal population have the potential to be particularly valuable, as human studies involving children present even more challenges than those focused on adults. The included models demonstrate that pretreatment with GnRHa therapy does not modify the effects of subsequent T administration, which were similar to those described in adult models. Importantly, their results suggest promising potential for future fertility in this population where no human data exist.

## 7. Conclusions

Recent data indicate that the number of individuals identifying as TGD is increasing, particularly among the adolescent and young adult populations, many of whom desire future children [1]. As a result, it is increasingly important to elucidate the implications of GAHT for future fertility and fertility-preservation procedures after GAHT. While not directly translatable to human medicine, animal models are easy to reproduce and manipulate and provide the ability to perform studies that would not be ethically permissible in humans. Additionally, animal models offer the ability to quickly assess potential impacts on subsequent generations born from GAHT-exposed gametes, such as oocytes cryopreserved while continuing GAHT. Although rodent models have proven invaluable for rapidly advancing our understanding of fertility after GAHT, other new animal models—particularly those with menstrual cycles—may add additional insight. Insights from animal models may eventually pave the way for human study and ultimately facilitate the development of robust, data-driven guidelines to improve healthcare for an increasingly gender-diverse society.

## 8. Future Directions

Regarding T-GAHT, all available studies administered hormonal treatments across a short timeframe. Future directions include the possibility of expanded studies to better understand the longer-term effects of T-GAHT on animal and, perhaps one day, human reproductive physiology. Animal models of pregnancy following T-GAHT washout are another promising area of research, as current human data remain limited to case studies and small reports. Future investigations into E-GAHT should be aimed at developing models that more closely mimic standard-of-care treatment regimens. From there, it will be important to delve beyond histological examination and begin to better understand the impact of feminizing GAHT on fertility outcomes and the interactions (if any exist) between treatment attributes such as dosing and duration with the extent of reversibility. Both available trials investigating GAHT in the peripubertal population utilized regimens that closely mimic human treatment regimens for puberty blockade and androgen therapy for transmasculine individuals. Their investigation of reproductive and IVF outcomes in their studies is novel. To our knowledge, no equivalent human studies exist in this area, precluding validation of these findings. The development of models of peripubertal feminizing GAHT is an additional area of future work. 

## Figures and Tables

**Table 2:** Animal models of fertility preservation in the setting of T-GAHT.

Study	Strain and Age	Hormone Treatment	Exposure and Washout Durations	Control(s)	Experimental Design(s)	Findings
Bartels et al., 2021 [24] Female mouse	Hsd:NSA (CF-1) 6 weeks	T cypionate Subcutaneous injection 400 ug weekly	6-week exposure 6–7-week washout	Exposure placebo: Sesame oil vehicle injections Stimulation control: Unstimulated	eCG COH + IVM or eCG COH + hCG ovulation induction. IVF and culture to 2-cell stage.	- ↓ Ovarian size - T mice responded to stimulation - ↑ Immature oocytes from active exposure - Similar numbers of oocytes from active exposure and washout - No difference in proportion of morphologically normal meiotic spindle formation - No difference in rates of fertilization or 2-cell embryos
Schwartz et al., 2023 [26] Female mouse	C57BL/6N 10 weeks	T enanthate Subcutaneous pellet implant 10 mg	Short-term 6-week exposure 2-week washout Long-term 12-week exposure 2-week washout	Sham pellet	COH + hCG ovulation induction. IVF and culture to blastocyst stage or transfer at cleavage stage.	COH + IVF during treatment: - Lower oocyte yield - No difference in maturity, fertilization, or blastulation COH + IVF after washout: - Short-term—Complete recovery - Long-term—Incomplete recovery

eCG: equine chorionic gonadotropin, hCG: human chorionic gonadotropin, COH: controlled ovarian hyperstimulation, IVM: in vitro maturation, IVF: in vitro fertilization.

**Table 3:** Animal models of E-GAHT.

Study	Strain and Age	Hormone Treatment	Exposure Duration	Control	Experimental Design	Findings
Alexander et al., 2022 [27] Male rat	Sprague Dawley 13 weeks	17-beta E2 benzoate Subcutaneous pellet implant 2.5 mg, 5 mg, or 7.5 mg	3 weeks	Sham pellet	Immediate post-treatment assessment of hormone profile and phenotypic and behavior changes	At all doses: - ↑ E2 to physiologic female levels - ↓ Testicular weight - ↓ Food intake, body weight, and lean mass
Pfau et al., 2023 [28] Male mouse	C57BL/6NHsd 8 weeks	Estradiol powder Subcutaneous pellet implant 1.25 mg, 2.5 mg, or 5 mg	6 weeks	Sham pellet	Immediate post-treatment assessment of hormone profile, phenotypic and behavior changes, sperm morphology, and testicular histology	- ↑ E2 to physiologic female levels - ↓ T and FSH—Suppression of LH at 5 mg dose - ↓ Testicular weight, size of the epididymis and seminal vesicles - ↓ Vacuolitic Sertoli cells at 2.5 mg and 5 mg doses - No changes in Leydig cells - Ongoing production of mature and immature spermatocytes - Abnormal spermatocyte motility
Tassarini et al., 2023 [29] Male rat	Sprague Dawley 9–10 week	17-beta E2 valerate + CPA Subcutaneous injection 0.09 + 0.33 mg, 0.09 + 0.93 mg, or 0.18 + 0.33 mg 5 times per week	2 weeks	Sesame oil injections	Immediate post-treatment assessment of hormone profile, phenotypic changes, and tissue histology	- ↑ E2 to physiologic female levels - Suppression of T - Significant systemic toxicity limits further generalizability
Gusmão-Silva et al., 2022 [30] Male rat	Wistar 2 months	E2 enanthate + DHPA Injection every 10 days Orchiectomized	5 months	Sham surgery; Sesame oil injection	Immediate post-treatment assessment of hormone profile and physiologic and phenotypic changes	- ↑ E2 to physiologic female levels - Suppression of T - Reduction in body weight and nasoanal length

FSH: follicle-stimulating hormone, LH: luteinizing hormone; CPA: cyproterone acetate; DHPA: dihydroxyprogesterone acetophenide.

## Data Availability

No new data were created during this study.
